# Dangerous Colors in Wall Papers

**Published:** 1878-03

**Authors:** 


### Selections.

### DANGEROUS COLORS IN WALL PAPERS.

Mr. L. Siebold, in a lecture on this subject, stated that out of sixty or seventy papers of various colors, blue, red, brown, pink, etc., analyzed by him, ten only were harmless, the rest containing arsenic. There is a popular impression that green papers are only to be feared; but the result of Mr. Siebold's examination should have the effect of rendering householders and heads of families suspicious of some of the most innocent looking colors. It is reasonable to assume that to the presence of deleterious ingredients contained in certain wall papers may be ascribed many little illnesses of children, where no apparent cause exists for the same, and which sometimes puzzle the medical attendant.

